# Living with Long COVID in a Southern State: A Comparison of Black and White Residents of North Carolina

**DOI:** 10.3390/ijerph22020279

**Published:** 2025-02-14

**Authors:** William Pilkington, Brooke E. Bauer, Irene A. Doherty

**Affiliations:** Julius L. Chambers Biomedical and Biotechnology Research Institute, North Carolina Central University, Durham, NC 27707, USA; wpilkington@ad.nccu.edu (W.P.);

**Keywords:** long COVID, minority populations, social determinants, disparities, lived experience

## Abstract

Long COVID can devastate patients’ overall quality of life, extending to economic, psychosocial, and mental health and day-to-day activities. Clinical research suggests that long COVID is more severe among Black and African American populations in the United States. This study examines the lived and lasting effects of long COVID among a diverse sample of North Carolina residents over one year by using three self-administered questionnaires completed online using Qualtrics. A cross-sectional descriptive analysis of the baseline results is presented. Our study recruited 258 adults, of which 51.5% had long COVID (but may have recovered), 32.3% had a COVID-19 infection at least once, and 16.3% had never had COVID-19. The socioeconomic status of Black participants was lower than that of White participants; however, the economic impact of long COVID was not worse. Across both groups, 64.4% were employed, 28.8% had to change tasks or work less, and 19.8% stopped working. Fewer White (32.6%) than Black (54.8%) participants always/often felt supported by family and friends about having long COVID. The majority of White participants (59.1%) reported that they did not recover from long COVID compared to 29.7% of Black participants. The long COVID/COVID-19 experience affected White and Black participants differently, but both populations continue feel the impacts.

## 1. Introduction

Since long COVID has been recognized as a separate disease from acute cases of COVID-19, vast research has been published on its all-encompassing clinical outcomes affecting nearly every organ system. The most recent definition developed by a committee of multidisciplinary experts convened by the National Academies of Sciences, Engineering, and Medicine defines it as “an infection-associated chronic condition that occurs after SARS-CoV-2 infection and is present for at least 3 months as a continuous, relapsing and remitting, or progressive disease state that affects one or more organ systems” [1]. Nationally representative studies have consistently estimated that 7% of adults in the United States (US) had long COVID during 2023–2024 [2,3,4]. A prospective study following 2524 patients for 12 months in Spain monitored symptoms and how SARS-CoV-2 variants manifested clinically. The prevalence of long COVID was similar across variants and specific genomic lineages, and both the Delta and Omicron variants were correlated with worse symptoms and disease severity [5]. Another analysis of electronic health records of over 441,000 US veterans showed that the incidence of long COVID did decrease during the pre-Delta, Delta, and Omicron periods from 10.42 cases/100,000 persons to 9.51/100,000 and 7.76/100,000 [6].

Racial and ethnic disparities of COVID-19 incidence and mortality in the United States emerged early during the pandemic, driven in part by ingrained adverse social determinants of health that persist in marginalized communities [7,8], including the periods when the Delta and Omicron variants predominated [9,10]. A review of electronic health records from over 62,000 patients in New York City reported that racial and ethnic minorities were more likely have symptoms and diagnoses consistent with long COVID [11].

In addition to the health effects, the financial and economic impact of long COVID has been analyzed at multiple levels. In a rigorous economic simulation, Bartsch et al. estimated the economic burden of long COVID in the United States, costing society at least USD 2.01–6.56 billion, employers at least USD 1.99–6.49 billion in productivity losses, and third-party payers USD 21–68.5 million annually [12]. An estimated two to four million Americans were unemployed in 2022 because of long COVID [13], while others who remained employed reduce their hours. Individuals already experiencing financial hardship have and will continue to experience increased food and housing insecurity due in part to long COVID’s associations with reduced income stability [14,15].

Long COVID also diminishes quality of life and relationships with others. It is associated with social isolation, limited energy, mental fatigue, and changes in personality that adversely affect marital relationships and friendships [16]. Additionally, a lack of long COVID societal awareness drives self-doubt and stigma among those affected, sometimes reinforced by interactions with health care workers [17]. Among patients in a prospective cohort study with mild COVID-19 who did not require hospitalization but experienced symptoms lasting longer than 6 months, the persistence of disease was significantly associated with long-term health status, a poorer quality of life, and psychological distress [18]. 

One small study that aimed to characterize the impact of long COVID on quality of life among Black and African Americans participants reported that their day-to-day roles, personal identity, and interpersonal relationships declined [19]. A mixed-methods analysis of long COVID symptoms among Black participants suggests that those living with long COVID experienced significantly more adverse mental health outcomes such as anxiety, depression, and hopelessness than those who had acute COVID-19 [20]. Black Americans face many structural barriers to long COVID treatment such as limited paid time off and physician referral pattern inefficiencies that make seeking treatment difficult [21].

This study aimed to describe the lived experiences of persons with long COVID and characterize and compare persons with long COVID to those who had on COVID-19 infections in North Carolina. Furthermore, because social and structural determinants of health disparities among racial and ethnic minorities persist in the southeastern United States, differences between Black and White populations were also examined.

## 2. Materials and Methods

### 2.1. Recruitment and Eligibility

To ensure that we reached people in marginalized communities as well as patients actively receiving treatment, we used several different approaches to recruit study participants. We did use a formal sampling strategy and framework to identify potential participants. Our initial goal was to recruit a study sample of 300 participants. However, our recruitment process was slower than expected. Flyers were posted in the long COVID clinic at the University of North Carolina Medical Center in Chapel Hill and other medical clinics throughout the state. Before COVID-19 tests were widely available, the Advanced Center for COVID-19 Related Disparities (ACCORD) at North Carolina Central University (NCCU) held numerous testing events in underserved communities across 15 counties. People who completed the survey at these events had the choice to give us permission to contact them for future studies. Everyone with an email address in the registry was sent an invitation with the link and QR code to the long COVID survey. Our researchers also distributed surveys at community health events in counties throughout the state, so people could register to receive an email with the link to the consent form and baseline survey. These events attracted hundreds of participants and afforded our researchers the opportunity to reach a sizable proportion of the minority population in the state. 

Eligibility criteria included being 18 years of age or older; having the ability to complete the survey in English; and being a resident of North Carolina with a valid postal address. To avoid the possibility of people completing the survey multiple times, we monitored the postal addresses and IP addresses closely. Duplicate responses were excluded from the dataset. Participants received a USD 50 gift card that was sent to validated North Carolina addresses. We did not limit eligibility to people who only had long COVID to allow for comparison. The survey was completed online using Qualtrics, beginning with the consent form and mailing address. Most questions were asked to the complete sample, with skip patterns for those who had COVID-19 and long COVID. The Institutional Review Board at North Carolina Central University approved the study.

### 2.2. Measures

The survey was self-administered electronically using Qualtrics. Respondents were free to skip any question or stop completing it altogether. They could either select “prefer not to answer” or simply skip any question. In either case, it was coded as missing.

The baseline survey assessed whether respondents had long COVID, had COVID-19 only, or had never had COVID-19 via the questions listed below. Long COVID (question 4) was assessed based on the definition of long COVID at the time the study was implemented using plain language.

(1) How many times have you had a COVID-19 infection, even if you were not tested?
  Never (not that I know of); 1 time; 2 times; 3 times or more; Prefer not to answer
(2) How long did your symptoms last after you first knew you had COVID-19? (If you had COVID more than once, pick the longest time your symptoms lasted).
  Less than a week; 1 to 2 weeks; 2 weeks to about a month; Between 1 to 3 months; Longer than 3 months
(3) Did you get new symptoms after recovering from your COVID infection(s)?
  Yes; No; Don’t know/not sure
(4) The disease known as Long COVID means having COVID symptoms that lasted for more than a month or 30 days OR getting new symptoms after the infection is over. Do you think that you have or had Long COVID?
  Yes; No; Don’t know/not sure; Prefer not to answer

Because 10.5% of respondents reported that they did not know if they had long COVID, we reclassified participants with long COVID on the basis of their responses regarding the duration of symptoms (question 2) and whether they experienced new symptoms after the acute disease (question 3). Specifically, a participant was reclassified as having long COVID if their answer to question 2 indicated that they had symptoms that lasted either between 1 and 3 months or longer than 3 month OR if they answered affirmatively to question 3 indicating that they experienced new symptoms after recovering from the initial infection.

Other questions assessed socioeconomic status, employment status, changes to employment, health insurance, and health care use and access, social support, Paxlovid use, vaccine uptake, and other chronic conditions unrelated to long COVID (e.g., type 2 diabetes, obesity, hypertension). 

**Figure 1:**
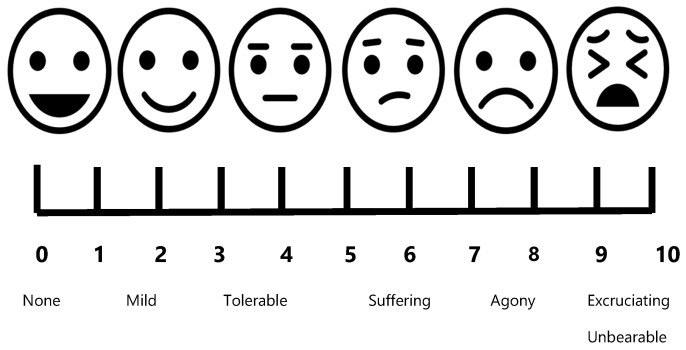
Adapted Wong–Baker FACES pain scale.

To capture quantitative data about the day-to-day struggles with common symptoms experienced by long COVID patients, we adapted the Wong–Baker FACES pain scale, a method originally developed for children to express how much pain they felt (Figure 1) [22]. The scale has been validated for measuring chronic pain and includes six faces on a scale from zero to ten with words below the numbers to assess how much they suffered over the last 30 days from overall physical pain, brain fog, breathing problems, physical exhaustion, stress, severe sadness, and depression.

We chose to limit our questions on the vast array of long COVID symptoms and illnesses because the clinical manifestations of long COVID have been widely studied in thousands of publications. This study aimed to describe the lived experience resulting from this disease and COVID-19 in general. 

### 2.3. Analysis

The analyses generated descriptive statistics and allowed us to compare characteristics across strata with parametric and nonparametric comparison tests as appropriate for continuous measures and frequencies for categorical data. Chi-square and Fischer’s Exact tests were computed for tabular comparisons. Fischer’s Exact tests were used when any of the cell sizes were less than 30. Given the small numbers, most tests were nonparametric. 

## 3. Results

### 3.1. Total Study Population

Two hundred fifty-eight people enrolled in the study. Half of the study participants (51.6%) had long COVID, 32.2% had COVID-19 at least once, but not long COVID, and 16.3% had never contracted COVID-19 (Table 1).

The distribution of participant characteristics shown in Table 1 was usually comparable by COVID-19 status (i.e., long COVID, COVID-19 only, no COVID-19 infections). Most study participants were Black (61.6%), followed by White (22.9%). We were unable to reach Latino communities; only five Latinos enrolled in the study. The mean age was 43 years (std dev = 14.9). Compared to national estimates, the proportions of respondents with chronic diseases were under-reported and therefore are not presented.

**Figure 2:**
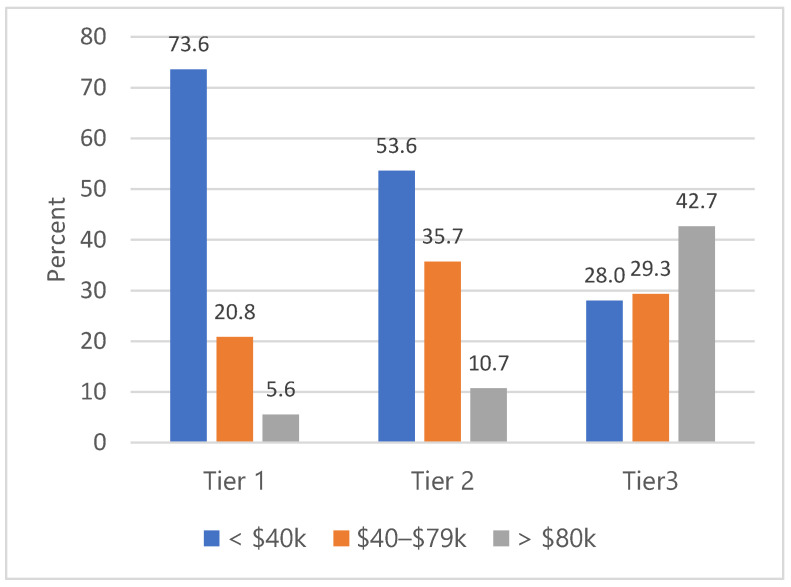
Income by county tier.

The participants resided in 35 of North Carolina’s 100 counties. The counties were approximately equally distributed by economic tier: 31.1%, 36.6%, and 32.3% of participants resided in Tier 1, Tier 2, and Tier 3 counties, respectively. As shown in Figure 2, the distribution of respondents’ annual income aligns with the tier of their county of residence. Most respondents living in Tier 1 counties (the lowest economic classification) (73.6%) earned < USD 40,000 annually, whereas the majority respondents in Tier 3 counties (42.7%) earned ≥ USD 80,000 annually.

### 3.2. Participants with Long COVID or COVID-19 Only

**Figure 3:**
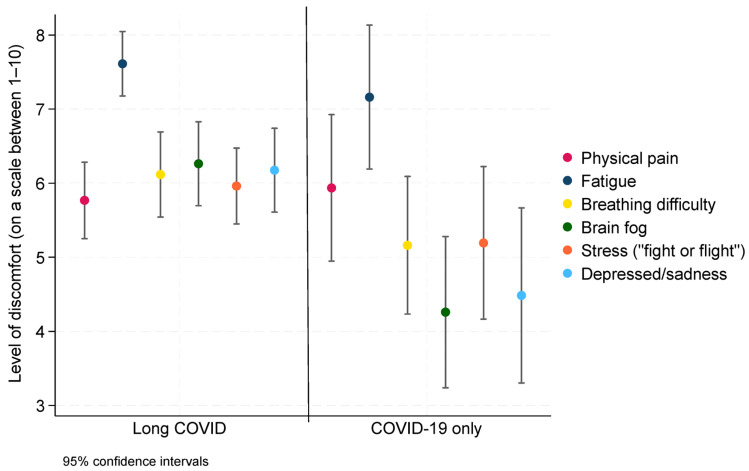
Mean values of levels of discomfort stratified by long COVID status.

Figure 3 presents the mean values (and 95% confidence intervals) from the adapted FACES pain scale among participants who had long COVID (*n* = 133, Table 1) or COVID-19 acute infections (*n* = 83, Table 1). Although the confidence intervals overlap between groups, the point estimates nevertheless suggest that the degree of discomfort was higher for some conditions among long COVID participants and comparable for both groups for other conditions. Among long COVID study participants, exhaustion and fatigue had the highest scores (7.61, 95% CI (7.18, 8.05)), which corresponds to between “suffering” and “unbearable” on the face scale, and they also experienced high levels of suffering from brain fog, difficulty breathing, and depression. Both groups reported values of approximately 6 for physical pain and fatigue/exhaustion, which corresponds to “suffering”.

### 3.3. Characteristics Among White and Black Participants with Long COVID or COVID-19 Only

Table 2 presents comparisons between White and Black respondents who had either long COVID or COVID-19; 33 participants were neither White nor Black and were excluded. Significantly more White (73.3%) than Black participants (57.3%) had long COVID (*p*-value = 0.033). One-quarter (25.4%) of this sub-sample did not know if they fully recovered. The majority of White participants reported that they did not recover (59.1%), which was significantly higher compared to Black participants (29.7%) (*p*-value = 0.004). 

The distribution of educational level and income in Table 2 shows socioeconomic disparities by race. Nearly 40% of Black participants had a high-school education at most and 33.3% received a college or graduate degree; in contrast, more than half of White participants had a college degree (30% bachelor, 23.3% graduate) (*p*-value = 0.005). Only 26.7% of White respondents compared to 48.1% of Black respondents earned less than USD 40,000 annually (*p*-value = 0.010). Comparable proportions of respondents in each racial group were employed (~60%). Also, as a result of their disease, at some point, 28.8% changed tasks at work and 19.4% stopped working.

Few respondents were uninsured, and 41.7% of White respondents compared to 26.7% of Black respondents had private insurance (*p*-value = 0.039), whereas significantly more Black (42%) than White (26.7%) respondents had public or government-funded health insurance (*p*-value = 0.042). Emergency room visits since contracting COVID-19 or long COVID were reported by significantly more Black (38.9%) than White (23%) participants (*p*-value = 0.045). Importantly, COVID-19 vaccine uptake was substantially higher among White (85.0%) than Black (69%) participants (*p*-value = 0.021).

We conducted a tabular analysis for the characteristics in Table 2 among the subset of Black and White participants who only had long COVID and found similar disparities.

### 3.4. Feelings and Activities Among Black and White Participants with Long COVID Only

The data collected about how long COVID changed lives are reflected in Table 3a,b. Specifically, Table 3 presents the results from the subset of 44 White respondents and 75 Black respondents who had long COVID. They experienced feelings of caution, regret, and being misunderstood about having long COVID (Table 3a). Less than half of respondents (40.7%) rarely felt that they had to be careful about who they told that they have long COVID and 46.1% rarely regretted disclosing they had it. Feeling misunderstood about having the hidden disability of long COVID, however, was more prevalent, because 37.1% and 25.9% of respondents felt this way sometimes or always/often, respectively. The most notable changes in daily life (Table 3b) are that long COVID impeded Black respondents from visiting family (*p*-value = 0.007) and spending time with friends (*p*-value = 0.057).

## 4. Discussion

This study aimed to better understand the effects and lived experiences of long COVID among North Carolina residents with a focus on comparing minority (i.e., Black) and non-minority populations (i.e., White). Examination of the data by race is extremely important; North Carolina is a state in the southeastern US where entrenched racism, injustices, and mistreatment of Black and African Americans endured for decades. A total of 258 people, recruited using several strategies, from 35 of the 100 counties in North Carolina enrolled. These results are not generalizable because of selection bias resulting from recruitment methods.

Instead of inventorying participants’ symptoms, as done in much of the literature, we assessed and examined the effects of living with this sometimes extremely debilitating disease. We adapted the Wong-Baker FACES pain scale [22] to assess how broad sets of symptoms impact the day-to-day lives of participants. Regardless of whether they had long COVID or only COVID-19 infections, participants suffered from substantial levels of pain (average ~6 out of a scale from 0 to 10). Brain fog among participants with long COVID was agonizing, and fatigue and exhaustion was excruciating. The combination of living with a lot of pain, in a mental brain fog, and constant exhaustion clearly quantifies the debilitating effects of long COVID.

In general, the socioeconomic disparities among Black participants stemming from racism and structural inequalities were present. Significantly more Black participants were undereducated with lower annual incomes. The impact of long COVID on employment status, however, was similar for both Black and White participants. Significantly fewer Black respondents reported receiving at least one COVID-19 dose. This is essentially consistent with our research conducted in similarly economically distressed communities before the vaccine was available; Black respondents were more likely to report COVID-19 vaccine hesitancy [23].

This study suggests that White patients may not recover from long COVID as quickly as Black patients, but this is based on self-reports without clinical data. Nevertheless, self-reported recovery among White participants was exceptionally low (18.2%), whereas, in contrast, almost half of the Black respondents reported that they had recovered.

Our study has limitations. First, selection bias and recruitment bias likely emerged as recruitment was primarily passive, with heavy reliance on people enrolling into the study through seeing the QR code or link for Qualtrics. HIPPA regulations prevented us from contacting patients directly who receive care at long COVID clinics. We also could not access sources such as statewide surveillance databases for the same reasons. The goal was to reach marginalized communities and often hidden populations wary of engaging with researchers after decades of mistreatment (e.g., the Tuskegee Syphilis experiment that lasted 40 years among Black men who were denied treatment after it became available). That said, the use of the registry of people from the other research studies at North Carolina Central University permitted us to reach people in historically marginalized communities. Although we did have 163 Black participants in our study (163/258), other minority populations were unrepresented in the subanalyses. 

## 5. Conclusions

Long COVID has had severe impacts on the physical and mental health and economic and social areas of affected populations, and our research confirms that these effects have been severe. Our findings further substantiate concerns that long COVID may last for years and prevent victims from working and performing normal daily activities. Our study results also suggest that employment impacts of long COVID may be more evenly distributed among Black and White populations than previously reported. These findings point to the need for more research on the effects of long COVID on all population groups and suggest that a large-scale longitudinal study may prove useful in helping us to better understand the susceptibility and severity of this debilitating malady. 

## Figures and Tables

**Table 1:** Demographic and socioeconomic status of long COVID study participants in North Carolina.

	Long COVID		COVID-19 Only		NO COVID-19		Total	
	*N*	Column%	*N*	Column%	*N*	Column%	*N*	
Total	133	(51.5)	83	(32.2)	42	(16.3)	258	
Race/ethnicity								
Asian	3	(2.3)	2	(2.4)	2	(4.8)	7	(2.7)
American Indian	3	(2.3)	7	(8.4)	3	(7.1)	13	(5.0)
Black	73	(54.9)	54	(65.1)	32	(76.2)	159	(61.6)
Latino	5	(3.8)	0		0		5	(1.9)
White	41	(30.8)	16	(19.3)	2	(4.8)	59	(22.9)
Other race	1	(0.8)	0		0		1	(0.4)
Multirace	6	(4.5)	2	(2.4)	0		8	(3.1)
Declined to report	1	(0.8)	2	(2.4)	3	(7.1)	6	(2.3)
Age	42.7		42.1		46.5		43.1	
(95% CI)	(40.3 45.0)		(38.7 45.5)		(41.5 51.5)		(41.3 45.0)	
Gender								
Woman	101	(76.5)	57	(68.7)	21	(50.0)	179	(69.6)
Man	28	(21.2)	26	(31.3)	20	(47.6)	74	(28.8)
Other	3	(2.3)	0		1	(2.4)	4	(1.6)
Marital status								
Married/cohabitating	55	(42.0)	26	(31.3)	12	(30.0)	93	(36.6)
Separated/divorced/widow	19	(14.5)	13	(15.7)	8	(20.0)	40	(15.7)
Single	57	(43.5)	44	(53.0)	20	(50.0)	121	(47.6)
Education								
≤High school/GED	35	(27.0)	28	(35.0)	23	(57.5)	86	(34.4)
Trade/some college	38	(29.2)	24	(30.0)	10	(25.0)	72	(28.8)
Bachelor degree	33	(25.4)	20	(25.0)	4	(10.0)	57	(22.8)
Graduate degree	24	(18.5)	8	(10.0)	3	(7.5)	35	(14.0)
Annual income								
<USD 40 k	58	(43.6)	38	(45.8)	24	(57.1)	120	(46.5)
USD 40–79 k	37	(27.8)	21	(25.3)	9	(21.4)	67	(26.0)
>USD 80 k	28	(21.1)	13	(15.7)	4	(9.5)	45	(17.4)
Not reported	10	(7.5)	11	(13.3)	5	(11.9)	26	(10.1)
Employment status								
Employed	81	(60.9)	57	(68.7)	21	(50.)	159	(61.6)
Unemployed	24	(18.0)	5	(6.0)	9	(21.4)	38	(14.7)
Not in work force	28	(21.1)	21	(25.3)	12	(28.6)	61	(23.6)
Changes in work status since pandemic or long COVID-19 (not mutually exclusive)								
Less time/changed tasks	48	(36.1)	17	(23.6)	7	(9.7)	72	(8.0)
Stopped working	33	(25.8)	8	(9.6)	2	(4.7)	43	(17.0)

**Table 2:** Comparison of White and Black participants with long COVID and COVID.

	White		Black		Total		Chi-sq or
	*N*	(Col %)	*N*	(Col %)	*N*	(Col %)	Fischer’s Exact
Total	60	(31.4)	131	(68.6)	191		*p*-Value
Long COVID							
Yes	44	(73.3)	75	(57.3)	119	(62.3)	0.033
No	16	(26.7)	56	(42.7)	72	(37.7)	
Recovered from LC (only LC participants)							
Yes	8	(18.2)	32	(43.2)	40	(33.9)	0.004
No	26	(59.1)	22	(29.7)	48	(40.7)	
Don’t know	10	(22.7)	20	(27.0)	30	(25.4)	
Gender							
Woman	45	(77.6)	94	(71.8)	139	(73.5)	0.402
Man	13	(22.4)	37	(28.2)	50	(26.5)	
Marital status							
Married/cohabitating	29	(48.3)	46	(35.4)	75	(39.5)	0.003
Separated/divorced/widow	15	(25.0)	16	(12.3)	31	(16.3)	
Single	16	(26.7)	68	(52.3)	84	(44.2)	
Education							
≤High school/GED	8	(13.3)	49	(39.9)	57	(30.6)	0.005
Trade/some college	20	(33.3)	34	(27.0)	54	(29.0)	
Bachelor degree	18	(30.0)	29	(23.0)	47	(25.3)	
Graduate degree	14	(23.3)	14	(11.1)	28	(15.1)	
Annual income							
<USD 40 k	16	(26.7)	63	(48.1)	79	(41.4)	0.010
USD 40–79 k	19	(31.7)	35	(26.7)	54	(28.3)	
>USD 80 k	20	(33.3)	20	(15.3)	40	(20.9)	
Not reported	5	(8.3)	13	(9.9)	18	(9.4)	
Current work status							
Employed	36	(60.0)	87	(66.4)	123	(64.4)	0.646
Unemployed	10	(16.7)	17	(13.0)	27	(14.1)	
Not in workforce	14	(23.3)	27	(20.6)	41	(21.5)	
Changes in work status since COVID-19							
Change tasks or work less	18	(30.0)	37	(28.2)	55	(28.8)	0.804
Stopped working	14	(23.3)	23	(17.6)	37	(19.4)	0.43
Health insurance (not mutually exclusive)							
None	3	(5.0)	13	(9.9)	16	(8.4)	0.254
Private	25	(41.7)	35	(26.7)	60	(31.4)	0.039
Public/government-funded	16	(26.7)	55	(42.0)	71	(37.2)	0.042
Purchased	9	(15.0)	16	(12.2)	25	(13.1)	0.596
Military	2	(3.3)	7	(5.3)	9	(4.7)	0.722
Other types	7	(11.7)	5	(3.8)	12	(6.3)	0.053
Been to the Emergency room since having long COVID or COVID-19							
Yes	14	(23.3)	51	(38.9)	65	(34.0)	0.045
No	46	(76.7)	79	(60.3)	125	(65.4)	
General health							
Excellent/very good	15	(25.0)	45	(35.2)	60	(31.9)	0.355
Good	24	(40.0)	47	(36.7)	71	(37.8)	
Fair/poor	21	(35.0)	36	(28.1)	57	(30.3)	
Received at least one dose of first COVID-19 vaccine							
Yes	51	(85.0)	87	(69.0)	138	(74.2)	0.021
No	9	(15.0)	39	(31.0)	48	(25.8)	
Obtained a flu shot in past 12 months							
Yes	38	(63.3)	64	(51.2)	102	(55.1)	0.120
No	22	(36.7)	61	(48.8)	83	(44.9)	

**Table 3:** (**a**) Feelings about having long COVID among White and Black participants with long COVID; (**b**) effects of long COVID on activities among White and Black participants with long COVID.

(a)							
	White		Black		Total		Fischer’s
	*N*	(%)	*N*	(%)	*N*	(%)	Exact
Total	44	(37.0)	75	(63.0)	119		*p*-Value *
Careful about who you tell that you have long COVID							
Rarely	20	(45.5)	28	(37.8)	48	(40.7)	0.529
Sometimes	12	(27.3)	28	(37.8)	40	(33.9)	
Always/Often	12	(27.3)	18	(24.3)	30	(25.4)	
Regret having told some people that you have long COVID							
Rarely	20	(45.5)	33	(46.5)	53	(46.1)	0.22
Sometimes	19	(43.2)	22	(31.)	41	(35.7)	
Always/Often	5	(11.4)	16	(22.5)	21	(18.3)	
Misunderstood for having long COVID, a “hidden” disability							
Rarely	16	(36.4)	27	(37.5)	43	(37.1)	0.47
Sometimes	14	(31.8)	29	(40.3)	43	(37.1)	
Always/Often	14	(31.8)	16	(22.2)	30	(25.9)	
Supported by friends and family about having long COVID							
Rarely	5	(11.6)	12	(16.4)	17	(14.7)	0.016
Sometimes	24	(55.8)	21	(28.8)	45	(38.8)	
Always/Often	14	(32.6)	40	(54.8)	54	(46.6)	
**(b)**							
	**White**		**Black**		**Total**		**Fischer’s**
	***N***	**(%)**	***N***	**(%)**	***N***	**(%)**	**Exact**
**Total**	**44**	**(** **37.0** **)**	**75**	**(** **63.0** **)**	**119**		***p*** -Value *
Doing regular household chores or other daily activities							
Rarely	4	(9.1)	9	(12.2)	13	(11.0)	0.282
Sometimes	22	(50.0)	46	(62.2)	68	(57.6)	
Always/often	16	(36.4)	17	(23.0)	33	(28.0)	
Not applicable	2	(4.5)	2	(2.7)	4	(3.4)	
Caring for children or other family							
Rarely	12	(27.3)	14	(18.9)	26	(22.0)	0.175
Sometimes	10	(22.7)	31	(41.9)	41	(34.7)	
Always/often	7	(15.9)	19	(25.7)	26	(22.0)	
Not applicable	15	(34.1)	10	(13.5)	25	(21.2)	
Visiting family							
Rarely	15	(34.1)	14	(18.9)	29	(24.6)	0.007
Sometimes	12	(27.3)	43	(58.1)	55	(46.6)	
Always/often	14	(31.8)	15	(20.3)	29	(24.6)	
Not applicable	3	(6.8)	2	(2.7)	5	(4.2)	
Spending time with friends							
Rarely	8	(18.2)	16	(21.6)	24	(20.3)	0.057
Sometimes	17	(38.6)	41	(55.4)	58	(49.2)	
Always/often	18	(40.9)	15	(20.3)	33	(28.0)	
Not applicable	1	(2.3)	2	(2.7)	3	(2.5)	
Working or being employed for pay							
Rarely	9	(20.5)	22	(30.1)	31	(26.5)	0.09
Sometimes	10	(22.7)	25	(34.2)	35	(29.9)	
Always/Often	18	(40.9)	17	(23.3)	35	(29.9)	
Not applicable	7	(15.9)	9	(12.3)	16	(13.7)	
Volunteering							
Rarely	8	(18.6)	18	(25.0)	26	(22.6)	0.005
Sometimes	9	(20.9)	29	(40.3)	38	(33.0)	
Always/often	17	(39.5)	10	(13.9)	27	(23.5)	
Not applicable	9	(20.9)	15	(20.8)	24	(20.9)	

* All comparisons exclude respondents who selected not applicable.

## Data Availability

To acquire access to the raw data supporting the conclusions of this article, please contact Irene A. Doherty (idoherty@nccu.edu) and Undi Hoffler Director, Research Compliance & Technology Transfer (uhoffler@nccu.edu) to apply for a data use agreement.
